# Non-Random Integration of the HPV Genome in Cervical Cancer

**DOI:** 10.1371/journal.pone.0039632

**Published:** 2012-06-27

**Authors:** Martina Schmitz, Corina Driesch, Lars Jansen, Ingo B. Runnebaum, Matthias Dürst

**Affiliations:** Klinik für Frauenheilkunde und Geburtshilfe, Universitätsklinikum Jena, Jena, Germany; Pontificia Universidad Catolica de Chile, Faculty of Medicine, Chile

## Abstract

HPV DNA integration into the host genome is a characteristic but not an exclusive step during cervical carcinogenesis. It is still a matter of debate whether viral integration contributes to the transformation process beyond ensuring the constitutive expression of the viral oncogenes. There is mounting evidence for a non-random distribution of integration loci and the direct involvement of cellular cancer-related genes. In this study we addressed this topic by extending the existing data set by an additional 47 HPV16 and HPV18 positive cervical carcinoma. We provide supportive evidence for previously defined integration hotspots and have revealed another cluster of integration sites within the cytogenetic band 3q28. Moreover, in the vicinity of these hotspots numerous microRNAs (miRNAs) are located and may be influenced by the integrated HPV DNA. By compiling our data and published reports 9 genes could be identified which were affected by HPV integration at least twice in independent tumors. In some tumors the viral-cellular fusion transcripts were even identical with respect to the viral donor and cellular acceptor sites used. However, the exact integration sites are likely to differ since none of the integration sites analysed thus far have shown more than a few nucleotides of homology between viral and host sequences. Therefore, DNA recombination involving large stretches of homology at the integration site can be ruled out. It is however intriguing that by sequence alignment several regions of the HPV16 genome were found to have highly homologous stretches of up to 50 nucleotides to the aforementioned genes and the integration hotspots. One common region of homologies with cellular sequences is between the viral gene E5 and L2 (nucleotides positions 4100 to 4240). We speculate that this and other regions of homology are involved in the integration process. Our observations suggest that targeted disruption, possibly also of critical cellular genes, by HPV integration remains an issue to be fully resolved.

## Introduction

A persistent infection with high risk human papillomaviruses (HR-HPV) in particular HPV16 and 18 is recognized as the highest risk factor for the development of cervical cancer [1]. Most HR-HPV infections are either latent or permissive. Latent infections are ill defined but it is assumed that the viral genome is maintained as an episome in the basal and parabasal cells of the epithelium without inducing obvious phenotypic alterations in the host cell. Virus replication in terms of virion production is confined to terminally differentiated cells of the intermediate and superficial epithelial layers and results from a switch of a latent to a permissive phase or directly from an acute infection. Normally HR-HPV infections are self-limited and resolve within several months. However, in an estimated 10% of cases a transforming type of HPV infection evolves. This transformation process is characterized by the deregulation of viral oncogenes E6 and E7 in cycling cells which ultimately results in chromosomal instability and the accumulation of mutations. The underlying mechanisms for deregulation are manifold. Integration of the HPV genome is a characteristic step in cervical carcinogenesis and its appearance correlates with the progression of precancerous lesions (CIN2/3) to invasive carcinoma [2], [3], [4], [5], [6]. However, integration is not mandatory in this process and was shown to be HPV-type dependent. Vinokurova and colleagues observed that HPV16, 18 and 45 were substantially more often present in an integrated state compared with HPV types 31 and 33 [7]. Interestingly the highest carcinogenic potential is ascribed to HPV16 and HPV18 [8].

The loss of the viral E2 gene is a common consequence of HPV integration. This event may lead to an elevated expression of the oncogenes E6 and E7 due to the fact that E2 is no longer able to repress the expression of the viral oncogenes *in trans* [9], [10]. However, of note is that in a recent analysis of biopsy material no correlation between the expression levels of viral oncogene transcripts and the physical state of the viral genome was found [11]. Since the transcriptionally active viral integration sites thus far analysed in CIN2/3 or cervical carcinomas represent the end point of a clonal selection process the most pragmatic interpretation of the data is that integration ensures a constitutive expression of the viral oncogenes at a level required to maintain the transformed state of the cell. More recently several investigators have also focussed on the impact integration may have on the host genome. A systematic analysis of the genome structure at the integration locus has revealed frequent genomic structural alterations at the HPV insertion sites in cervical carcinoma [12]. Two further publications provide evidence for a complete functional loss of the tumor suppressor genes, *ZBTB7C* and *CASZ1*, respectively. In both cases gene expression is prevented due to insertional mutagenesis in combination with loss of heterozygosity [13], [14]. Although such constellations are likely to be rare events it becomes increasingly more evident that HPV integration does not occur entirely at random. In a previous study we could show that the majority of viral-cellular fusion transcripts in cervical carcinomas co-transcribe cellular sequences of known or predicted genes. Indeed, 17 of 74 (23%) of the integration sites were located within the cytogenetic bands 4q13.3, 8q24.21, 13q22.1, and 17q21.2, in clusters ranging from 86 to 900 kb. The integration hotspots 8q24.21 and 13q22.1 are close to adjacent fragile sites. Of interest is that integration within the MYC locus on 8q24.21 is strongly correlated with high levels of *MYC* expression [15], [16], [17] implying *cis* regulatory effects being exerted by the viral genome.

The phenomenon of non-random integration of HPV DNA is intriguing and may not just be a question of chromatin accessibility in transcriptionally active regions or fragile sites which are prone to double strand breaks [18], [19], [20]. For a better understanding of the role and mechanisms of virus integration in cervical carcinogenesis it is necessary to gather more data for comparative purposes. Accordingly, we have analysed the viral-cellular fusion transcripts of 34 HPV16 positive and 13 HPV18 positive cervical carcinomas. On the basis of this new data the previously defined hotspots for integration could be confirmed and extended upon. There is also mounting evidence that numerous genes are affected more than once by integration.

## Results

In 47 of 87 HPV16 or HPV18 positive tumors analysed viral-cellular fusion transcripts could be amplified. Fusion transcripts were detected more frequently in HPV18 positive tumors (72%) than in HPV16 positive tumors (49%). The remaining tumors contained either only episomal viral genomes or integrated HPV DNA which is transcriptionally silent. The cellular sequences of the fusion transcripts were characterized using the NCBI human megaBlast tool. To identify expressed sequence tags and predicted genes all additional cellular sequences were analysed using the UCSC blat database.

### Chromosomal Assignment of the Fusion Transcripts

**Figure 1:**
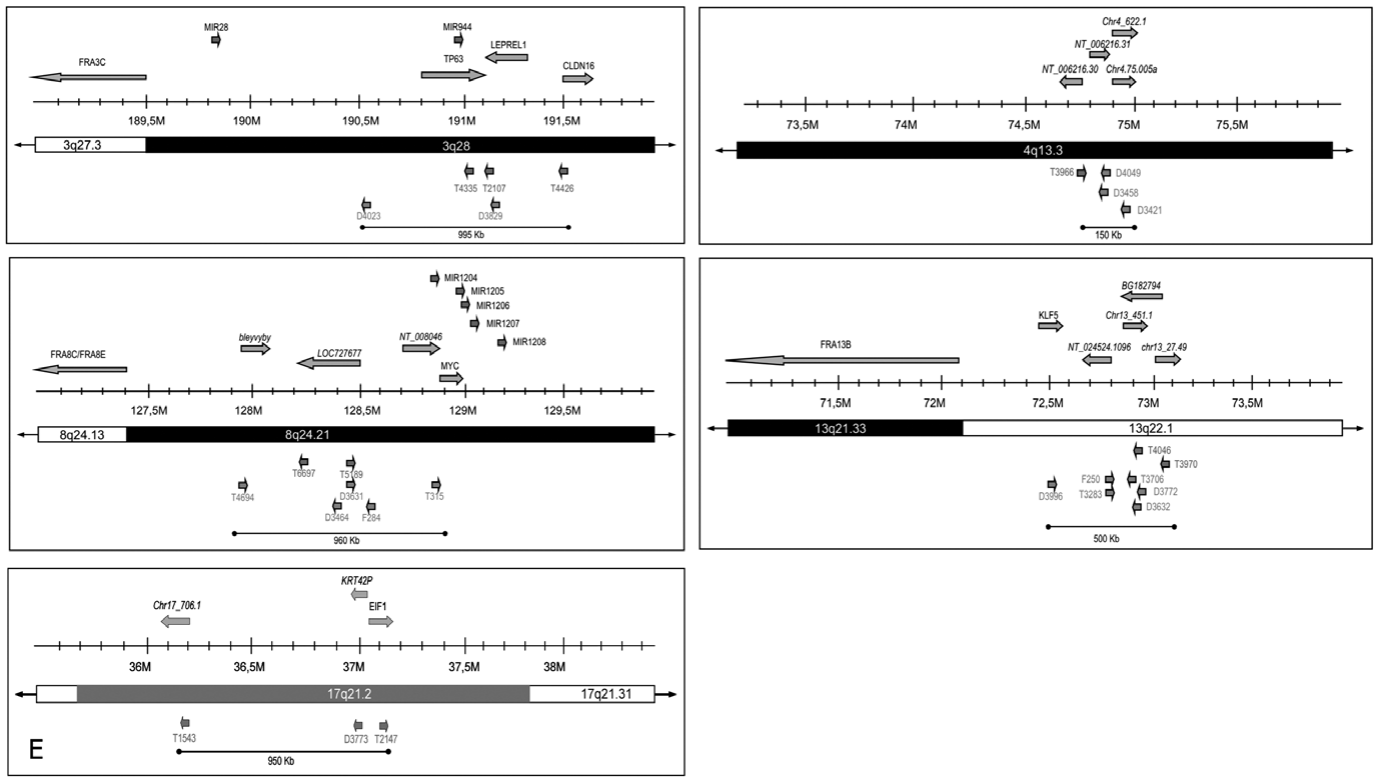
Chromosomal hotspots for HPV integration. Depicted are integration sites located within the cytogenetic bands 3q28 (A), 4q13.3 (B), 8q24.21 (C), 13q22.1 (D) and 17q21.2 (E). Light blue arrows: genes affected by HPV integration; red arrows: HPV fusion transcripts described in this work; grey arrows: HPV fusion transcripts described by Kraus et al. 2008; green arrows: fragile site; dark blue arrows: microRNAs.

In total, 23 fusion transcripts contained sequences of known genes and 23 contained sequences of predicted genes and ESTs. In one case the cellular sequence did not match any database entries. The viral cellular fusion transcripts could be assigned to all chromosomes, except for chromosome 11, 14, 16, 18 and 20 and are summarized in table 1. In a previous study we had already described that some regions are more frequently affected by integration than other parts of the genome [21]. For three of these hotspots we have now found additional integration events: The regions 8q24.21 and 13q22.1 were affected twice and region 4q13.3 was affected once. Moreover, three fusion transcripts were mapped to a 600 kb region in the chromosomal cytogenetic band 3q28 and thus represent a new hotspot for HPV integration. Figure 1 includes the data from Kraus and colleagues and depicts all five hotspots, their size and the vicinity to related fragile sites and miRNAs.

**Table 1:** Summary of all viral-cellular fusion transcripts analysed.

Sample	HPV	Chr. match	Accession Code	Gene Name	Spliced or fused to	Orientation	Fragile site^1^	miRNAs	
T3726	16	1p36.21	C1orf196	Chromosome 1 open reading frame 196	n.a.	AS	FRA1A (1p36)	-	
T6248	18	1p36.22	intergenic		-		FRA1A (1p36)	MIR34a: 9,211,727–9,211,836MIR5697: 10,027,439–10,027,516MIR1273d: 10,287,776–10,287,861	1p36.22
T2319	16	1p36.22	CASZ1	Castor zinc finger 1	exon 21	S	FRA1A (1p36)		
T4347	16	1q42.3	BC016972 ( = uc001hwk.1)		n.a.	AS	FRA1H (1q42.1) 5 Mb distance	MIR4753: 235,353,349–235,353,431MIR1537: 236,016,300–236,016,360MIR4428: 237,634,419–237,634,491	1q42.3
T1900	18	1q42.3	LYST	Lysosomal trafficking regulator	n.a.	AS	FRA1H (1q42.1) 5 Mb distance		
T5066	16	2p16.3	NRXN1	Neurexin 1	n.a.	AS	FRA2D (1p16.2) 1 Mb distance	-	
T892	16	2p23.2	GPN1	GPN-loop GTPase 1	n.a.	AS	-	MIR1301: 25,551,509–25,551,590MIR4263: 28,219,234–28,219,316	2p23.32p23.2
T1875	16	2q33.1/2q32.3/2q33.1	ORC2/intergenic/BZW1	Origin recognition complex, subunit 2/basic leucine zipper and W2 domains 1	n.a.-exon 1	AS-S	-	-	
T2882	16	2q22.3	N-SCAN: chr2.3.305.a		intron 6–7	S	*FRA2K (2*q*22.3)*		
T3562	18	2q22.3	N-SCAN: chr2.3.305.a		n.a.	AS	*FRA2K (2*q*22.3)*		
T3987	16	2q34	ERBB4	V-erb-a erythroblastic leukemia viral oncogene homolog 4 (avian)	n.a.	ASAS	FRA2I (2q33) 3 Mb distance	MIR548f2: 213,290,987–213,291,084MIR4776: 213,790,981–213,791,060	2q34
T5234	16	3p21.31	USP4	Ubiquitin specific peptidase 4 (proto-oncogene)	intron 15–16	S	-	MIR425: 49,057,581–49,057,667 MIR191: 49,058,051–49,058,142MIR4271: 49,311,553–49,311,619MIR5193: 49,843,570–49,843,678MIR566: 50,210,759–50,210,852MIR4748: 50,712,511–50,712,594	3p21.31
T18	16	3q26.2	MDS1	MDS1 and EVI1 complex locus	n.a.	AS	-	MIR551b: 168,269,642–168,269,737 MIR569: 170,824,453–170,824,548	3q26.2
T4335	16	3q28	TP63	Tumor protein p63	n.a.	AS	FRA3C (3q27) 2 Mb distance	MIR28: 188,406,569–188,406,654 MIR944: 189,547,711–189,547,798	3q28
T4426	16	3q28	Gencode: CLDN16		n.a.	AS	FRA3C (3q27) 2 Mb distance		
T2107	16	3q28	LEPREL1	Leprecan-like 1	exon 15intron 14–15	SS	FRA3C (3q27) 2 Mb distance		
T458	16	4p14	Genscan: NT_006238.1		intron 14–15	S	FRA4D (4p15) 5 Mb distance	MIR5591: 39,413,530–39,413,594MIR4802: 40,504,057–40,504,136	4p14
T3966	16	4q13.3	Genscan: NT_006216.30		n.a.	AS	-	-	
T4024	16	4q23	C4orf17/LOC84103	Chromosome 4 open reading frame 17	n.a.	AS	-	MIR3684: 99,918,538–99,918,611	4q23
T106	16	4q31.21	Genscan: chr4_71.7		n.a.	AS	FRA4C (4q31.1) 200 Kb distance	-	
T4034	18	5q13.2	RGNEF	Guanine nucleotide exchange factor	exon 37	S	-	-	
T3377	18	5q23.1	BC036311		exon 3	S	-	MIR1244-2: 118,310,281–118,310,365MIR5706: 118,490,332–118,490,411	5q23.1
T3576	16	6p21.1	N-SCAN: chr6.1.607.a		exon 2	S	-	MIR4647: 44,221,943–44,222,022MIR4642: 44,403,378–44,403,459MIR586: 45,165,411–45,165,507	6p21.1
T2128	16	6p25.1	Genscan: chr6_3.24N-SCAN EST: chr6.7.003.a		intron 1–2n.a.	SAS	FRA6B (6p25.1)	-	
T4502	18	7q34	UBN2	Ubinuclein 2	exon 13	S	-	MIR4468: 137,808,504–137,808,567	7q33
T5189	16	8q24.21	LOC727677		n.a.	AS	FRA8C/*FRA8E* 8q24.1 1 Mb distance	MIR1205: 128,972,879–128,972,941 MIR1206: 129,021,144–129,021,202 MIR1207: 129,061,398–129,061,484 MIR1204: 128,808,208–128,808,274 MIR1208: 129,162,362–129,162,434	8q24.21
T6697	18	8q24.21	LOC727677		exon 6	S	FRA8C/*FRA8E* 8q24.1 1 Mb distance		
T1509	16	9p13.3	UBAP2	Ubiquitin associated protein 2	n.a.	AS	-	-	
T5254	18	9p21.3	N-SCAN: chr9.132.a		n.a.	AS	FRA9C/FRA9D (9p21)	MIR491: 20,716,104–20,716,187MIR31: 21,512,114–21,512,184MIR4474: 20,502,263–20,502,340MIR4473: 20,411,146–20,411,236	9p21.3
T4601	16	9p23	N-Scan: chr9.068.aGenscan: NT_008413.226		intron 8–9n.a.	SAS	-	-	
T186e	16	9q21.32	Genscan: NT_023935.256		intron 2–3	S	-	MIR7-1: 86,584,663–86,584,772	9q21.32
T3256	16	10q24.2	Geneid: chr10_1119.1		intron 1–2	S	*FRA10A (10*q*24.2)*	MIR608: 102,734,742–102,734,841	10q24.31
T1820	16	12q21.33	EST: AA296845		n.a.	AS	FRA12B (12q21.3)	-	
T4046	16	13q22.1	Geneid: chr13_451.1		n.a.	AS	FRA13B (13q21) 800 Kb distance	-	
T3970	18	13q22.1	Genscan: chr13_27.49		n.a.	AS	FRA13B (13q21) 1 Mb distance	-	
T182e	16	15q22.1	LIPC	hepatic Lipase	exon 5	S	FRA15A (15q22)	MIR2116: 59,463,382–59,463,461	15q22.2
T1907	16	15q22.1	LIPC	hepatic Lipase	exon 9	S	FRA15A (15q22)		
T2317	16	15q26.1	SEMA4B	Sema domain, immunoglobulin domain (Ig), transmembrane domain (TM) and short cytoplasmic domain, (semaphorin) 4 B	n.a.	AS	-	MIR7-2: 89,155,056–89,155,165MIR3529: 89,155,078–89,155,155MIR9-3: 89,911,248–89,911,337 MIR1179: 89,151,338–89,151,428MIR5094: 90,393,869–90,393,953MIR5009: 90,427,163–90,427,262MIR3174: 90,549,987–90,550,073	15q26.1
T3427	16	17p11.2	Ensemble: ENST00000431878Genscan: chr17_10.56		exonn.a.	SAS	*FRA17A (17*q*23.1)* 4 Mb distance	MIR1180: 19,247,819–19,247,887	17p11.2
T4112	18	17p13.1	FXR2	Fragile X mental retardation, autosomal homolog 2	exon 8	S	*FRA17A (17*q*23.1)* 3 Mb distance	MIR497: 6,921,230–6,921,341MIR324: 7,126,616–7,126,698MIR195: 6,920,934–6,921,020	17p13.1
T2723	18	17q23.1	TMEM49	Transmembrane protein 49	exon 12	S	FRA17B (17q23.1)	MIR454: 57,215,119–57,215,233 MIR301a: 57,228,497–57,228,582MIR4729: 57,443,444–57,443,515MIR21: 57,918,627–57,918,698MIR4737: 58,120,386–58,120,466	17q22
T3231	18	17q23.1	TMEM49	Transmembrane protein 49	exon 12	S	FRA17B (17q23.1)		17q23.1
T2967	16	19p13.11	GATAD2A	GATA zinc finger domain containing 2A	exon 9	S	*FRA19B (19p13)*	MIR640: 19,545,872–19,545,967 MIR1270–1: 20,510,081–20,510,163MIR1270–2: 20,579,240–20,579,322	19p13.11
T4292	16	19p13.12	NANOS3	Nanos homolog 3 (Drosophila)	exon 3	S	*FRA19B (19p13)*	MIR693: 14,640,355–14,640,452 MIR181C: 13,985,513–13,985,622 MIR181D: 13,985,689–13,985,825 MIR23a: 13,947,401–13,947,473 MIR27a: 13,947,254–13,947,331MIR24-2: 13,947,101–13,947,173MIR5684: 12,897,942–12,898,006MIR5695: 13,031,134–13,031,218	19p13.12
T4995	18	19p13.3	CNN2	Calponin 2	exon 7	S	*FRA19B (19p13)*	MIR4745: 804,940–805,001MIR3187: 813,584–813,653MIR1227: 2,234,061–2,234,148 MIR1909: 1,816,158–1,816,237MIR4321: 2,250,638–2,250,717	19p13.3
T3840	16	21q11.2	EST: DR423233		n.a.	AS	-	MIR99a: 17,911,409–17,911,489 MIRlet7c: 17,912,148–17,912,231 MIR125B2: 17,962,557–17,962,645	21q21.1
T4417	16	Xp21.3	ARX	Aristaless related homeobox	n.a.	AS	-	-	

1 Common and rare fragile sites located at a distance of up to 5 Mb adjacent to the integration locus. Rare fragile sites are shown in italics.

‡ n.a.: not applicable, because fusion transcript is in antisense orientation.

### Association with Fragile Sites and miRNAs

Of the 47 integration loci identified, 10 (21%) occurred within a common fragile site (CFS) and 6 (13%) in rare fragile sites. Moreover, in 15 (32%) cases the integration sites were flanking fragile sites at a distance of 200 kb up 5 Mb. Another 16 integration sites were not associated with fragile sites. Moreover, 32 integration sites were located within a distance of 3 Mb to miRNAs (table 1).

### Orientation of ORFs within Fusion Tanscripts

Twenty-three of 47 fusion transcripts contained sequences of known genes. In 12 cases the host gene was orientated in the direction of viral promoter i.e. both viral and human sequences were in sense orientation. In almost all of these tumors, the viral sequence was spliced to a cellular exon sequence (11 of 12 events). 23 fusion transcripts contained predicted gene sequences and 8 were integrated in sense orientation. In 3 cases the orientation could not be determined because of inconsistency among the databases used (table 1).

### Identical Genes are Affected by Integration in different Tumors

By compiling the data of this study and published data five genes and four predicted genes could be identified which were affected at least twice in independent tumors (Table 2). Four of these genes are located in the hotspots 3q28 (*LEPREL1* and *TP63*), 8q24.1 (*LOC727677*) and 13q22.1 (*BG182794*). The other genes are located elsewhere in the genome. Whereas the genes *Chr2.3.305.a*, *LEPREL1*, *NT_008046.7*, and *LIPC* were affected twice by integration, *TP63* was affected three times, *LRP1B*, *LOC727677* and *BG182794* four times and *TMEM49* even six times.

**Table 2:** Genes affected by HPV integration at least twice in individual tumors.

Name	HPV	Pathology	Locus	Gene	Gene size (nt)	Spliced to‡	Orientation	Detection method	Reference^†^
D3918	18	CxCa	2q22.1	LRP1B	1.900.274	n.a.	AS	Apot	Kraus 2008
T654	45	CxCa	2q22.1	LRP1B		n.a.	AS	Apot	Kraus 2008
T19	16	CxCa	2q22.1	LRP1B		exon 28	S	Apot	Ziegert 2003
HK9	18	CxCa	2q22.1	LRP1B		intron 41^2^	n.s.	Restriction PCR	Ferber 2003
T2882	16	CxCa	2q22.3	chr2.3.305.a	567.027	intron 6–7	S	Apot	
T3562	18	CxCa	2q22.3	chr2.3.305.a		n.a.	AS	Apot	
D3829	45	CxCa	3q28	LEPREL1	164.391	exon 9	S	Apot	Kraus 2008
T2107^1^	16	CxCa	3q28	LEPREL1		exon 15	S	Apot	
				LEPREL1		intron 14–15	S	Apot	
T4335	16	CxCa	3q28	TP63	265.852	n.a.	AS	Apot	
Clone C5	16	cell line	3q28	TP63^3^		intron^2^	n.s.	Restriction PCR	Dall 2008
int4	16	VINX	3q28	TP63		n.s.	n.s.	Apot	Wentzensen 2002
D4045	18	CxCa	8q21.2	NT_008046.7	5.247.020	intron 2	S	Apot	Kraus 2008
D4056	45	CxCa	8q21.2	NT_008046.7		intron 2	S	Apot	Kraus 2008
D3464	18	CxCa	8q24.21	LOC727677^4^	192.322	exon 6	S	Apot	Kraus 2008
D3631	45	CxCa	8q24.21	LOC727677^4^		n.a.	AS	Apot	Kraus 2008
T5189	16	CxCa	8q24.21	LOC727677		n.a.	AS	Apot	
T6697	18	CxCa	8q24.21	LOC727677		exon 6	S	Apot	
T3706	16	CxCa	13q22.1	BG182794	133.369	intron 1	S	Apot	Kraus 2008
D3772	18	CxCa	13q22.1	BG182794		intron 1	S	Apot	Kraus 2008
D3632	45	CxCa	13q22.1	BG182794		exon 2	S	Apot	Kraus 2008
T4046	16	CxCa	13q22.1	BG182794		exon 2	S	Apot	
T182e	16	CxCa	15q22.1	LIPC	136.898	exon 5	S	Apot	
T1907	16	CxCa	15q22.1	LIPC		exon 9	S	Apot	
T2723	18	CxCa	17q23.1	TMEM49	133.087	exon 12	S	Apot	
T3231	18	CxCa	17q23.1	TMEM49		exon 12	S	Apot	
T30	18	CxCa	17q23.1	TMEM49^5^		exon 2	n.s.	Apot	Ziegert 2003
T24	18	CxCa	17q23.1	TMEM49^5^		exon 3^2^	AS	Dips	Ziegert 2003
107	16	CxCa	17q23.1	TMEM49^6^		exon 12^2^	n.s.	Restriction PCR	Thorland 2003
200229	16	CIN2	17q23.1	TMEM49^5^		n.s.	n.s.	Dips	Matovina 2009

‡ n.s.: not specified; n.a.: not applicable, because fusion transcript is in antisense orientation; ^†^integration sites without reference result from this work.

1 Two fusion transcripts were found in T2107; ^2^refers to sequenced integration sites; ^3^TP73L alias TP63; ^4^ *sweeker* in Kraus 2008 is no longer listed in any database; ^5^VMP1 alias TMEM49; ^6^DKFZP566I133 alias TMEM49.

Moreover, the genes *NT_008046.7*, *LOC727677*, *BG182794* and *TMEM49* are of particular note because at least two tumors harbour identical viral cellular fusion transcripts with respect to the viral donor and acceptor splice sites used.

## Discussion

HPV integration into the host genome is likely to be a very frequent event but cannot be readily detected if integration occurs in a single cell without subsequent clonal selection pressure. In most cervical cancers there is only one transcriptionally active HPV integration site. There is evidence that these integration sites represent early clonal events which have provided a selective advantage for the expansion of the neoplasm. Because of their contribution to the carcinogenic process elucidation of these particular integration events are pertinent for understanding HPV-induced carcinogenesis.

Viral-cellular fusion transcripts are molecular markers for transcriptionally active integration sites and can be detected by a 3̀RACE protocol (the APOT assay) which allows PCR amplification for subsequent sequence analysis. In this study we have identified viral-cellular fusion transcripts in 47 cervical carcinomas. With one exception the fusion transcripts comprise cellular sequences of either known or predicted genes. This is in line with the results of our previous analysis and suggests that integration occurs mostly in transcriptionally active regions [21]. Moreover, in agreement with the literature 66% of the integration events were either within (34%) or adjacent to a fragile site (32%) [18], [19], [20], [21]. Also integration of HPV DNA near miRNAs is evident. miRNAs are associated with the regulation of important processes such as development, proliferation, differentiation and apoptosis [22] and they are often deregulated in cancer cells [22], [23]. Of the 75 miRNAs in the neighborhood of integration sites 19 have already been associated with cancer (figure 1). Of these miR-34a, miR-191, miR-28, miR-944, miR-31, miR-7-2, miR-9-3, miR-497, miR-195, miR-301a, miR-21, miR-181c, miR-27a, miR-99a and miR-let7c are expressed in cervical cancer cells [24], [25], [26], [27], [28], [29]; whereas miR-34a, miR-21, miR-191, miR-9-3, miR-181d, miR-23a, miR-24-2, miR-99a and miR-125b2 are reported to be involved in other tumor entities [28], [30], [31], [32].

Of note is that for a substantial number of fusion transcripts (11/47) the viral sequences are spliced in sense orientation to cellular exons of known genes. Disruption of a gene by HPV integration, even if this occurs within intron sequences, is likely to have an impact on gene expression and in rare instances was shown to have contributed to a complete loss of gene function [13], [14].

**Figure 2:**
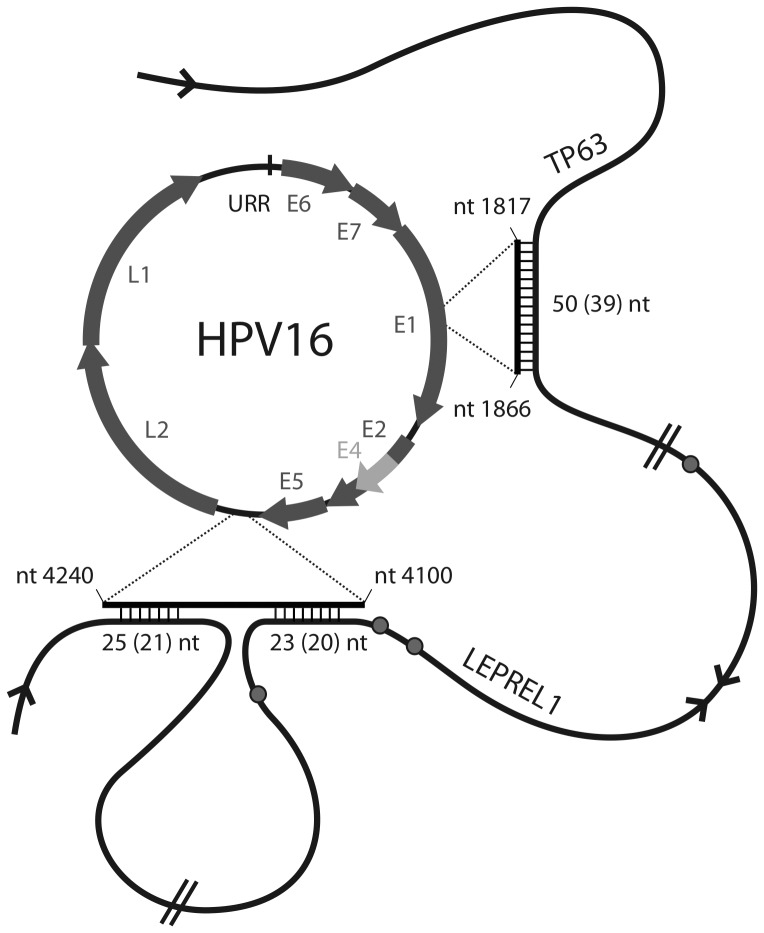
Sequence homologies between the HPV16 genome and the two adjoining genes *TP63* and *LEPREL1*. Three homologous stretches of up to 50 nucleotides are shown. The number of exact nucleotide matches is given in brackets (see also Sequences S1). The DNA loop between the two homologies located on *LEPREL1* comprises 102.689 nucleotides; the second loop 269.968 nucleotides. Grey dots refer to the approximate location of the corresponding viral-cellular fusion transcripts detected in tumors D3829, T2107 and T4335 (from left to right).

An important finding of this study is that it provides further support for a clustering of HPV integration sites in chromosomal hotspot regions. Of the 121 tumors analysed (including the data by Kraus et al., 2008) 22% of the viral-cellular fusion transcripts were assigned to one of five hotspots (figure 1). The hotspots range in size from 150 kb to 995 kb and contain up to 8 integration sites. Moreover, by including further published data 9 genes were found to be affected at least twice by HPV integration, four of these genes are located within the hotspots of the cytogenetic bands 3q28, 8q24.21 and 13q22.1 (table 2). This observation is of particular interest because it suggests that integration is not only associated with transcriptionally active regions and nearby fragile sites. Especially since in some tumors even identical viral-cellular fusion transcripts with respect to viral-cellular splicing were found (table 2). However, sequencing of the integration sites in case *TMEM49* had shown that the breakpoints of the two respective tumors are about 17 kb apart. Moreover, both integration sites showed no homology between the viral and cellular sequences [14]. DNA recombination involving large stretches of homology at the integration site can at least be ruled out for *TMEM49*. Intriguingly sequence alignment of the genes listed in Table 2 has revealed several regions with high homology to the HPV16 genome. The most common region of high homology is between the viral gene E5 and L2 (nucleotides positions 4100 to 4240). Six of the 9 genes affected at least twice by HPV integration show a minimum of 80% homology in stretches of up to 34 nucleotides (Sequences S1). Moreover, each of the 9 genes show further homologies with other parts of the viral genome. Exemplarily the positions of the homologous regions of the adjoining genes *LEPREL1* and *TP63* with the HPV16 genome are depicted in figure 2. We speculate that these regions may be involved in the integration process. It is envisaged that the viral genome is tethered to chromatin by Brd4 which plays a key role in chromosomal functional events such as transcription, DNA replication, repair and recombination [33], [34]. The homologous stretches of DNA may then allow annealing of partially dissociated strands and thereby contribute to the recombination event. Sequence homology at the integration site itself need not be a prerequisite. Beyond this highly speculative scenario it is also interesting to note that all of the genes involved are cancer associated genes to varying degrees. If functionally impaired, they may have played a role in the clonal selection process. For the predicted genes *chr2.3.305.a* (N-SCAN), *NT_008046.7*, *LOC727677* (alias sweeker) and *BG182794* no functional data are available. However, *LRP1B* belongs to the low density lipoprotein receptor gene family and plays a role in the process of receptor-mediated endocytosis. A homozygous loss of *LRP1B* in several cervical tumors was found using array-based comparative genomic hybridization analysis [35]. Also in other tumor entities like thyroid cancer [36], gastric cancer [37], lung cancer [38], [39], [40], [41] and oral cancers [42], [43] silencing or loss of *LRP1B* was observed. In functional terms *LRP1B* is able to inhibit cell migration [44] and its loss may thus be relevant for invasion and metastasis. The second gene, *LEPREL1*, encodes a protein involved in collagen biosynthesis, folding and assembly. Thus far this gene was not associated with cervical cancer but it was observed to be silenced breast cancer cell lines and in 26% of breast cancers analysed [45]. Two further genes are *TP63* and *LIPC*. *TP63*, a member of the p53 protein family, is another gene affected by integration. It acts as a tumor suppressor protein and aberrant expression was noted for several cancer entities including cervical cancer [46], [47]. *LIPC* is a cytoplasmic protein mainly expressed in the liver. It is involved in the lipidprotein metabolism and catalyzes hydrolysis of phospholipids, mono-, di- and triglycerides and acyl-CoA thioesters [48]. A direct link to carcinogenesis is not readily apparent but in a recent study a complete lack of expression was observed in 18% of cervical carcinomas. By contrast all normal cervical epithelia, metaplasia and CIN examined expressed LIPC [14]. The most frequently affected gene, being disrupted 6 times, is *TMEM49* (transmembrane protein 49), also known as *VMP1* (vacuole membrane protein 1) located on chromosome 17q23.1. It encodes a plasma membrane protein which is an essential component of initial cell-cell contacts and tight junction formations [49]. Reduced expression of *TMEM49* was found for invasive breast cancer cell lines and in kidney cancer metastasis [49]. *TMEM49* also appears to be particularly prominent in terms of deregulation of nearby miRNAs. miR21 is located only 676 bp downstream of this gene and may as a consequence of HPV integration be either up or down-regulated. Association of this miRNA has been reported for the caspase cascade [50] and it can target BTG2, a gene with antiproliferative properties [51]. Thus far miR21 was shown to be upregulated in multiple cancers such as breast, lung, colon, pancreas, prostate, stomach, ovary and uterus [28], [31], [32].

By characterising an increasing number of transcriptionally active HPV integration sites it has become evident that integration is not an entirely random event but also involves preferred chromosomal sites. In individual cases this may impair gene function and promote malignant progression. Presently we can only speculate on the mechanisms that contribute to this phenomenon.

## Materials and Methods

### Clinical Samples

In this study, 69 HPV16 and 18 HPV18 positive biopsies from cervical carcinoma patients were screened for HPV integration by the APOT assay. For HPV genotyping a Taqman based multiplex real-time PCR assay was used [52]. All biopsies were taken from patients treated at the Department of Gynecology of Friedrich-Schiller-Universität, Jena, Germany between 1995 and 2008. All tumors were histopathologically classified as squamous cell carcinomas.

### Nucleic Acid Isolation

Total RNA was isolated using the NucleoSpin RNA II Kit (Macherey-Nagel, Düren, Germany) according to the protocol for RNA isolation from tissues. Samples were homogenized by the use of injection needles with a diameter of 0,55 mm. DNA was removed in all samples by DNase treatment for 15 min (RT). DNase was supplied with the NucleoSpin RNA II Kit. Total RNA was eluted in 60 µl RNase-free water and stored at −80°C for further analysis.

### Reverse Transcription

Total RNA (300–500 ng) was reverse-transcribed using 200 units of Superscript II reverse transcriptase (Invitrogen, Carlsbad, California, USA) and an oligo(dT) primer coupled to a linker sequence (5′-AAG CAG TGG TAT CAA CGC AGA GTA CT_(30)_VN-3′), referred to as CDS-Primer (Clontech, Heidelberg, Germany). The reaction was incubated for 70 min at 42°C in a final volume of 20 µl according to the protocol of the Superscript II Kit. 40 units of RNaseOUT (Invitrogen, Carlsbad, California, USA) were added to inhibit RNase activity.

### Amplification of Papillomavirus Oncogene Transcript (APOT) Assay

HPV-derived fusion transcripts were amplified using the APOT assay [6]. It is based on a 3′-rapid amplification of cDNA ends (RACE) performed in a nested PCR format. HPV E7 primers are used as forward primers (HPV16: first primer: CGG ACA GAG CCC ATT ACA AT, second primer: CCT TTT GTT GCA AGT GTG ACT CTA CG; HPV18: first primer: TAG AAA GCT CAG CAG ACG ACC, second primer: ACG ACC TTC GAG CAT TCC AGC AG) and an adapter primer complementary to the linker sequence in the CDS primer as first reverse primer and the CDS Primer as second nested primer.

The APOT assay was done as previously described [6] with slight modifications with regard to the primer used. The reverse primer for the first PCR comprises the sequence 5′-AAG CAG TGG TAA CAA CGC A-3′, the nested PCR primer the sequence 5′-AAG CAG TGG TAA CAA CGC AGA GTA CT-3′. The reaction mixture, containing 20 mM Tris-HCl, 50 mM KCl, 1.5 mM MgCl_2_, 200 µM dNTPs, 250 nM primer each and 0.75 units recombinant Taq Polymerase (Invitrogen, Carlsbad, California, USA), was subjected to an initial denaturation step for 5 min at 94°C, followed by 30 cycles of denaturation at 94°C for 30 sec, primer annealing for 30 sec and elongation at 72°C for 2 min. For HPV16, annealing temperatures of 61°C and 66°C for the first and second PCR, respectively, were used; for HPV18, 61°C and 68°C. The reaction was terminated by a final elongation step at 72°C for 6 min. Two microliters of the first PCR were used as template for the nested PCR step. Both reactions were performed in a volume of 25 microliters. The amplification products were visualized by 1% agarose gel electrophoresis. Products which differ in their size from the major viral transcript (E6*1–E7–E1_v_E4–E5) are indicative for viral-cellular fusion transcripts. The APOT assay has several limitations. A tumor in which the integrated HPV genome is transcriptionally silent will not reveal the characteristic viral-cellular fusion transcript and will therefore be scored as negative for integration. Moreover, the assay may not detect viral-cellular fusion transcripts in the presence of an excess of transcripts derived from episomal HPV genomes. Finally, a tumor in which the integrated HPV genome persists in form of a concatemer the viral transcripts may not comprise cellular sequences and therefore cannot be differentiated from episome-derived transcripts. Overall the assay underestimates the number of tumors with integrated HPV DNA.

### Sequence Analyses

Viral-cellular fusion transcripts were excised from the gel and extracted using the Zymoclean Gel DNA Recovery Kit (AnalytikJena, Jena, Germany). The isolated products were sequenced (Seqlab, Göttingen, Germany) and the integration locus was determined by database alignments using National Centre for Biotechnology Information (NCBI) human megaBlast tool and the University of California, Santa Cruz (USCS) genome browser.

### PCR

For selected samples (n = 13) the existence of viral-cellular fusion transcripts was verified by PCR using viral and cellular integration specific primers. PCR amplification was performed in a final volume of 25 microliters containing 20 mM Tris-HCl, 3 mM MgCl_2_, 50 mM KCl, 200 µM dNTPs, primer 400 nM each and 1.5U Platinum Taq DNA Polymerase (Invitrogen, Carlsbad, California, USA). PCR reactions were performed with an initial denaturation step at 94°C for 10 min, followed by 45 cycles of denaturation at 94°C for 15 sec, annealing at 58–60°C (depending on the primer pair used) for 20 sec and elongation at 72°C for 30 sec.

### In Silico Analyses

For chromosomal mapping of the viral-cellular fusion transcripts and to relate HPV integration to fragile sites and miRNAs, the University of California, Santa Cruz (UCSC) genome browser (hg19) and the Map Viewer (Build 37.3) of the National Centre for Biotechnology Information (NCBI) were used. All known miRNA sequences are listed in the miRNA registry “miRBase” (http://www.mirbase.org/) with 1527 records (release 18) for homo sapiens.

### Investigation of Integration Loci Frequency

To determine the frequency of HPV integration in specific genes and hotspots, data published by Dall 2008, Ferber 2003, Kraus 2008, Matovina 2009, Peter 2006, Thorland 2003, Wentzensen 2002 and 2004 and Ziegert 2003 [3], [15], [17], [19], [20], [21], [53], [54], [55] were included.

## Supporting Information

Sequences S1 **Sequence alignments of homologous regions.**
(DOC)
