# Supplementary material for: Non-Random Integration of the HPV Genome in Cervical Cancer
Source: PLoS One. 2012 Jun 27;7(6):e39632. doi: 10.1371/journal.pone.0039632 (PMC3384597; doi:10.1371/journal.pone.0039632)
Supplement: Sequences S1 — Sequence alignments of homologous regions. (DOC) [file pone.0039632.s001.doc]

**Supporting information – Sequences S1**

Matches between sequences of the frequently affected genes (table 2) and HPV 16 region between E5 and L2.

The first row comprises gene sequences, the second row HPV16 sequences.

BG182794

Gene 34934 CAATTTAATAAAAA-CAAACAAACAAA 34959

|| |||| |||||| ||||||||||||

HPV16 4221 CAGTTTATTAAAAAACAAACAAACAAA 4195

Gene 34936 ATTTAATAA-AAACAAACAAACAAAA 34960

||| || || ||||||||||||||||

HPV16 4215 ATTAAAAAACAAACAAACAAACAAAA 4190

Chr2.3.305.a

Gene 370335 tttttttGTTCCCTTGTTTGTTTTGTAATAAA 370366

|||||||||| ||||||||||| |||||||

HPV16 4187 TTTTTTTGTTTGTTTGTTTGTTTTTTAATAAA 4218

Gene 14550 AAACAAACAAACAAATAACAATTAAAAAAT 14579

||||||||||||||| || || |||||||

HPV16 4209 AAACAAACAAACAAACAAAAA--AAAAAAT 4182

Gene 530990 CAATGATTTTATGTACATATACAATGTGAAA 531020

|||| | ||||||||||||||| | || ||

HPV16 4122 CAATTACATTATGTACATATACATTATGTAA 4092

LEPREL1

Gene 114924 TACATATTGAAATCAAAAAGAAAAA 114948

|| |||| |||| |||||||||||

HPV16 4181 TATATATGAAAATAAAAAAGAAAAA 4157

Gene 12213 TTGTTTGTTTGTTTTTCTTGTAA 12235

|||||||||||||| | || |||

HPV16 4192 TTGTTTGTTTGTTTGTTTTTTAA 4214

LIPC

Gene 48254 ATTTCtttttttGTTAGTTTGTTTGTTT 48281

|||| |||||||||| ||||||||||||

HPV16 4182 ATTTTTTTTTTTGTTTGTTTGTTTGTTT 4209

Gene 1303 TTTGTTTGTTTACTTTTTAATGAAGT-TTATAAC 1335

||||||||||| |||||||| || | |||| ||

HPV16 4195 TTTGTTTGTTTGTTTTTTAATAAACTGTTATTAC 4228

LOC727677

Gene 73058 TTTTTTGTTTGCTTATGTGTTTATT 73082

||||||||||| || | ||||| ||

HPV16 4188 TTTTTTGTTTGTTTGTTTGTTTTTT 4212

LRP1B

Gene 161494 TTTCATATATAACttttttttttttt 161519

|||||||||||| |||||||||| ||

HPV16 4171 TTTCATATATAATTTTTTTTTTTGTT 4196

Gene 1860699 TTGTTTTCTATGATAACTTAATATTTTTTCTTT 1860731

||||| | || |||||||| ||||||||||||

HPV16 4133 TTGTTGTATACCATAACTTACTATTTTTTCTTT 4165

Gene 325295 AAAACAAACAAATTTaaaaaaaaaaCAAtt 325324

|||||||||||| | |||||||| ||||

HPV16 4210 AAAACAAACAAACAAACAAAAAAAAAAATT 4181

Gene 630037 ATTTACAAACAAGCAAACAAACAAA 630061

||| | |||||| ||||||||||||

HPV16 4215 ATTAAAAAACAAACAAACAAACAAA 4191

TP63

Gene 67501

ATTCTATTGGTATAAAACA-GAATAACAGACATGAATGCAAGTATATTGAG 67550

||| ||||||||||||||| | ||| || | || | || |||| ||| |||

HPV16 1817

ATTATATTGGTATAAAACAGGTATATCAAATATTAGTG-AAGTGTATGGAG 1866
